# Effect of ivabradine on cognitive functions of rats with scopolamine-induced dementia

**DOI:** 10.1038/s41598-022-20963-5

**Published:** 2022-10-10

**Authors:** Abdel-Azim Assi, Sara Abdelnabi, Abdelraheim Attaai, Rasha B. Abd-ellatief

**Affiliations:** 1 Department of Pharmacology, Faculty of Medicine, Assiut University, Assiut, Egypt; 2 Department of Anatomy and Embryology, Faculty of Veterinary Medicine, Assiut University, Assiut, 71526 Egypt

**Keywords:** Drug discovery, Neuroscience, Anatomy, Neurology

## Abstract

Alzheimer’s disease is among the challenging diseases to social and healthcare systems because no treatment has been achieved yet. Although the ambiguous pathological mechanism underlying this disorder, ion channel dysfunction is one of the recently accepted possible mechanism. Hyperpolarization-activated cyclic nucleotide-gated (HCN) channels play important roles in cellular excitability and synaptic transmission. Ivabradine (Iva), an HCN blocker, is acting on HCN channels, and is clinically used for angina and arrhythmia. The current study aimed to investigate the therapeutic effects of Iva against scopolamine (Sco) induced dementia. To test our hypothesis, Sco and Iva injected rats were tested for behavioural changes, followed by ELISA and histopathological analysis of the hippocampus. Induced dementia was confirmed by behavioural tests, inflammatory cytokines and oxidative stress tests and histopathological signs of neurodegeneration, multifocal deposition of congo red stained amyloid beta plaques and the decreased optical density of HCN1 immunoreactivity. Iva ameliorated the scopolamine-induced dysfunction, the hippocampus restored its normal healthy neurons, the amyloid plaques disappeared and the optical density of HCN1 immunoreactivity increased in hippocampal cells. The results suggested that blockage of HCN1 channels might underly the Iva therapeutic effect. Therefore, Iva might have beneficial effects on neurological disorders linked to HCN channelopathies.

## Introduction

Dementia is the most common neurodegenerative disease from which the Alzheimer’s disease (AD) is the most common among the elderly. AD is characterized by neuropsychiatric symptoms such as progressive memory impairment, personality changes, cognitive dysfunction, and language disorders, which seriously disturb the patient’s quality of life^1^. Rather than acute inflammation, the brain in Alzheimer's disease is characterised by gradual memory loss, chronic inflammation with an oxidative stress accompanied by higher levels of inflammatory cytokines^2,3^.

The affected population is increasing rapidly for unknown reasons. Although the huge efforts to advent a medication, no effective cure for AD has been discovered yet^4^. Since Alzheimer's disease was first identified at the beginning of the twentieth century, diagnosing it has been extremely difficult^5^. The molecular and clinical events, including amyloid accumulation, neuroinflammation, tau accumulation, neural degeneration, cognitive impairment, and behavioural psychological symptoms, develop along with AD progression^6^.

The HCN channels are members of the voltage-gated pore loop channel family. They play an important role in the generation of neuronal and cardiac automaticity^7^. The HCN channels are encoded by four genes (HCN1–4). These four subtypes of HCN channels, can be assembled into different combinations and conformations^8^. In the voltage range of activation, HCN channels carry an inward current, termed I_f_ in the heart and I_h_ in neurons. I_h_ currents, independent of synaptic plasticity changes mediated by *N*-methyl-d-aspartate (NMDA) receptor-dependent excitability changes, appear to modulate neuronal excitability^9^. HCN channels are widely distributed in the hippocampus, the associative cortices, and subcortical structures and may participate in the etiology or treatment of AD by affecting neuronal excitability and regulating Aβ generation^10–12^.

Ivabradine (Iva) is a broad-spectrum HCN cation blocker, which blocks the HCN-mediated current. It is already in use as a heart-rate-reducing agent in the clinic^12^. Recently, Iva has been shown to have central effects such as anticonvulsant, antioxidant, and neuroprotective properties^13^ and is effective at reducing seizure susceptibility^14^. Iva had an inhibitory effect, not only, on neuropathic pain, but also on inflammatory responses^15^. So, Iva’s effect may be important in the initial stage of inflammation. Furthermore, it was reported that Iva has anticonvulsant and neuroprotective effects against PTZ- and PICRO-induced seizures. It reveals a significant antioxidant effect in the prefrontal cortex (PFC), hippocampus and striatum (ST) by determination of MDA, GSH and nitrite levels. Also, it markedly downregulated the apoptotic marker (cleaved caspase-3) in the hippocampus^13^.

Donepezil, a selective and reversible AChE inhibitor^16^, that has been validated to have neurosupportive effects against many neurodegenerative diseases^17^ and improves the cholinergic and cognitive functions of patients with AD. It was the first-line anti-AD treatment medication and it has been shown to be effective and safe^18^. In view of the relationship between HCN channels and neuronal excitability in the modulation of cognitive function, the present study hypothesized that Iva could possess a therapeutic effect against Sco models of dementia, based on HCN blockage mechanisms in cerebral areas related to cognitive functions. We used Donepezil as a positive control, to compare the effects of administration of high and low dose of Iva, because it improves cognition and/or behavior, and is FDA approved for use for AD patients, although, does not alter the progression of the AD^17,19^.

## Results

### Validation of the Sco induction of dementia

#### Behavioural results

##### Passive avoidance test

**Figure 1:**
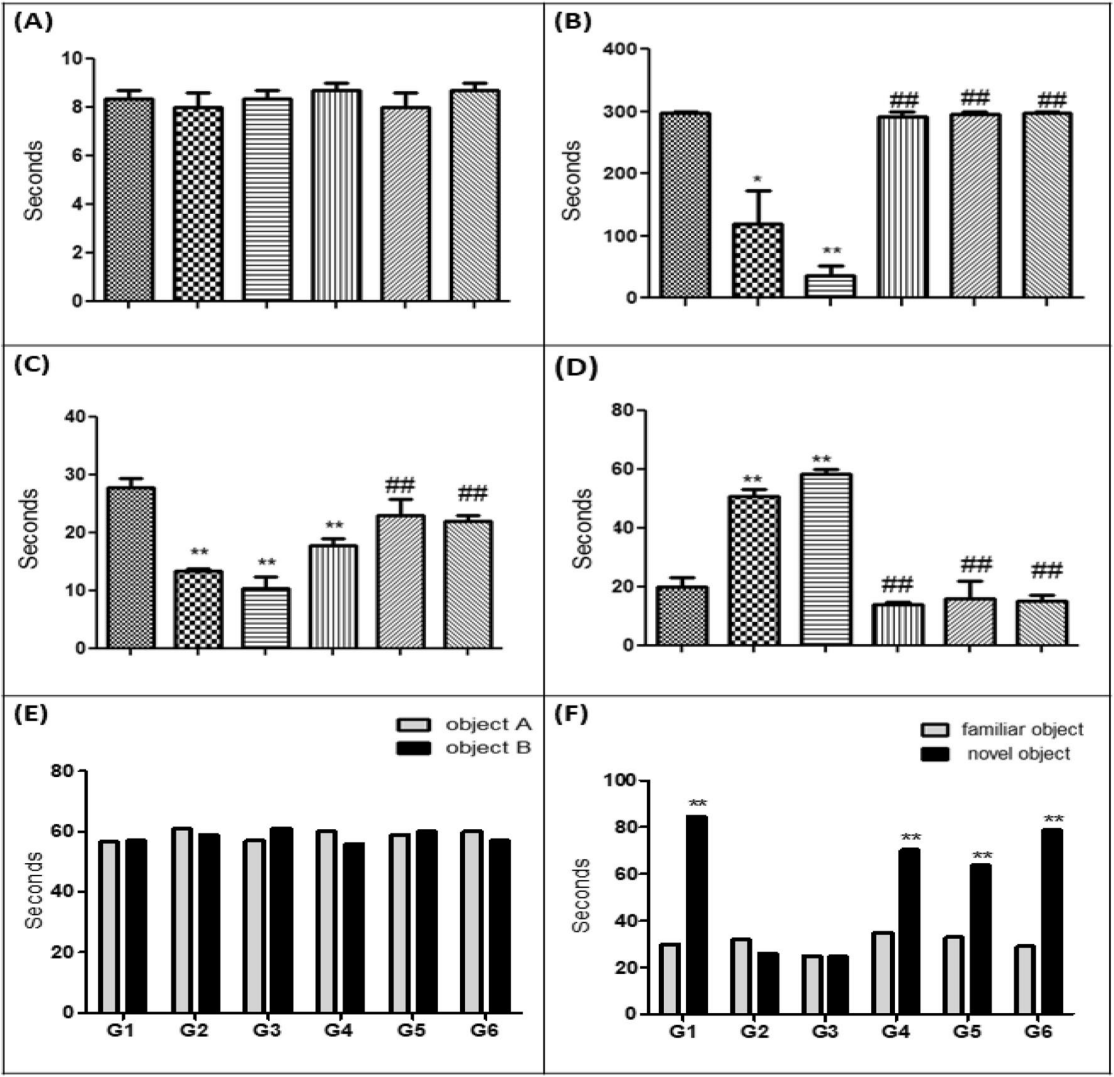
Analysis of behavioral tests: (**A**) Effect of ivabradine on time taken to enter the dark compartment in passive avoidance test in the first day which presented as IL in scopolamine induced dementia (in seconds). (**B**) Effect of ivabradine on time taken to enter the dark compartment in passive avoidance test which presented as STL in scopolamine induced dementia (in seconds). *PA* passive avoidance, *IL* initial latency, *STL* step-through latency. (**C**) Effect of ivabradine on time spent on the target quadrant where the platform was located during the probe trial in seconds in scopolamine induced dementia. (**D**) Effect of ivabradine on time taken to locate the position of the platform is presented as escape latency during the probe trial in seconds in scopolamine induced dementia (in seconds). (**A**–**C**) Data have been analyzed by using one way ANOVA p < 0.01. (**E**) The effect of ivabradine on the time spent exploring objects in the sample phase in scopolamine induced dementia in seconds. Values are analyzed using by two way ANOVA P = 0.8532 and expressed as mean ± SEM. of (8–10) rats. (**F**) The effect of ivabradine on the time spent exploring objects during novel object recognition test in scopolamine induced dementia in seconds. **Highly significant (P > 0.01) difference from familiar object. For all tests values are expressed as mean ± SEM of (8–10) rats. Stars were used for *significant (P > 0.05) and **highly significant (P > 0.01) difference from control group. Other symbols were used for ^#^significant (P > 0.05) and ^##^highly significant (P > 0.01) difference from dementia control group.

Rats of different groups didn't show a significant difference in initial latency (IL) during the acquisition phase (Fig. 1A). On the contrary, in the retention trial, Sco rats and ES rats demonstrated markedly less step through latency (STL) than controls (Fig. 1B). After 2 weeks of low and high (5 and 10 mg/kg/day for 2 weeks, respectively) Iva treatment, as well as in the standard AD medication, donepezil (0.5 mg/kg/day for 2 weeks) treatment, rats displayed significant increase in STL compared to ES rats (Fig. 1B). These rats memorized that their presence in the darkroom was associated with an aversive stimulus (foot shock). Moreover, the prolonged escape latency to the position of the formerly submerged platform significantly reduced in a manner comparable to the effect of donepezil.

##### Morris water maze test

Rats of different experimental groups showed a gradual decrease in the mean escape latency (to the hidden platform) throughout the 6 successive days of the acquisition trials, reflecting acquisition (learning) (Table 1). During the probe trial, ES rats showed less time spent in the target quadrant in search for the missing platform, after notably longer latencies to find the position of the escape platform, vs. control rats (Fig. 1C,D). Likewise, administration of high Iva and donepezil to ES group significantly attenuated the decrease in the time spent in the target quadrant on the 7th day, indicating memory retrieval (Fig. 1C,D). The only exception is the lower dose of Iva, whose effect on escape latency is significant and the time spent in the target quadrant was non-significant (Fig. 1C,D).

**Table 1:** Effect of ivabradine on escape latencies throughout the 6 successive days of the acquisition trials in Morris water maze in scopolamine induced dementia (in seconds).

Group	Treatment	Escape latency during the acquisition trial (s)					
		Day 1	Day 2	Day 3	Day 4	Day 5	Day 6
I	Saline	84.00 ± 3.06	74.00 ± 2.08	53.00 ± 4.16	41.33 ± 1.86	37.33 ± 2.186	26.67 ± 3.33
II	Scopolamine 6 mg/kg/day for 21 days	85.00 ± 2.04	75.75 ± 4.97	61.50 ± 6.46	61.25 ± 10.48	58.00 ± 1.225**	52.50 ± 4.787**
III	Scopolamine 4 mg/kg/day for 28 days	89.00 ± 0.58	78.75 ± 0.63	76.25 ± 0.75*	73.25 ± 1.18*	72.50 ± 1.041**	72.33 ± 2.33**
IV	Group III + ivabradine 5 mg/kg/day for 15 days	82.32 ± 2.33	71.00 ± 3.79	56.00 ± 2.08^#^	41.67 ± 7.27^#^	36.67 ± 3.333^##^	28.33 ± 6.01^##^
V	Group III + ivabradine 10 mg/kg/day 15 days	89.00 ± 1.00	71.67 ± 3.28	49.33 ± 4.33^##^	38.33 ± 1.67^##^	31.00 ± 3.055^##^	23.33 ± 4.41^##^
VI	Group III + donepezil 0.5 mg/kg/day 15 days	86.50 ± 0.87	70.25 ± 1.70	45.75 ± 3.25^##^	37.50 ± 3.23^##^	33.75 ± 2.394^##^	26.67 ± 3.33^##^

Values are expressed as mean ± SEM. of (8–10) rats. Analyzed using by one way ANOVA (p < 0.01). *Significant (P > 0.05) and **highly significant (P > 0.01) difference from control group. ^#^Significant (P > 0.05) and ^##^highly significant (P > 0.01) difference from dementia control group.

##### Novel object recognition time

As shown in (Fig. 1E), there was no significant differences in exploration time between object A and object B (same size and form) among all experimental groups. Sco and ES groups were unable to distinguish the familiar object and the novel object (Fig. 1F). Significant increase in novel object recognition time were restored after treatment with donepezil and both doses of iva (Fig. 1F).

### ELISA

**Figure 2:**
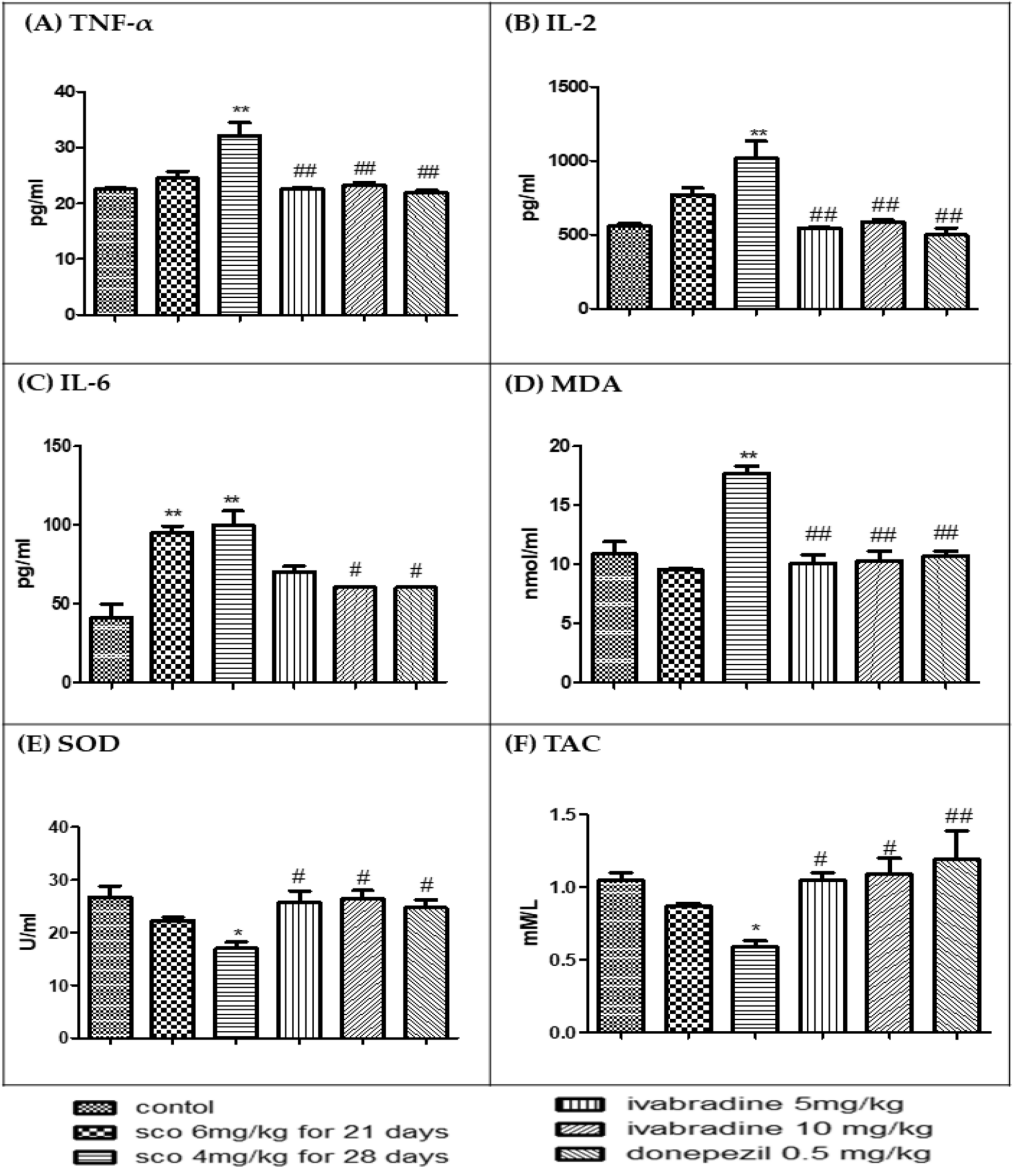
Analysis of inflammatory cytokines and oxidative stress: (**A**) Effect of ivabradine on hippocampal TNF-α level of scopolamine treated rats. (**B**) Effect of ivabradine on hippocampal inflammatory cytokines (IL-2) level of scopolamine treated rats. (**C**) Effect of ivabradine on hippocampal inflammatory cytokines (IL-6) level of scopolamine treated rats. (**D**) Effect of ivabradine on hippocampal lipid peroxidase (MDA) level of scopolamine treated rats. (**E**) Effect of ivabradine on hippocampal superoxide dismutase (SOD) level of scopolamine treated rats. (**F**) Effect of ivabradine on hippocampal total antioxidant capacity (TAC) level of scopolamine treated rats. *Significant (P > 0.05) difference from control group and ^#^Significant (P > 0.05) difference from dementia control group.

Compared to normal control, ES group demonstrated significantly higher hippocampal levels of inflammatory cytokines such as TNF-α, IL-2 and IL-6 (Fig. 2A–C). This high cytokine level was coupled with significant increase in Lipid peroxidase (MDA) level in ES group when compared to control group (Fig. 2D). Additionally, there was significant decrease in Superoxide dismutase (SOD) and total antioxidant capacity (TAC) level in ES group when compared to control group (Fig. 2E,F).

The analysis of the hippocampal homogenate using ELISA technique validated the behavioural results. There was significant decrease in the elevated level of TNF-α, IL2 and IL-6 in low and high Iva and donepezil when they compared to ES group (Fig. 2A–C). Moreover, there was a significant decrease in the level of MDA in low and high Iva groups and donepezil when they compared to ES group (Fig. 2D). Additionally, there was a significant increase in the level of SOD and TAC in low and high Iva and donepezil when they compared to ES group (Fig. 2E,F).

The brain and hippocampal size: In Group II (administrated Sco 6 mg/kg/day for 21 days) and Group III (ES group 4 mg/kg/day for 28 days): there was a decrease in the weight of the brain in group II and group III, marked decrease in group III, when compared with that of the control (Table 2). Moreover, there was a significant decrease in the cross-sectional area (CSA) of the sagittal-parasagittal sections of the Brain excluding cerebellum (BEC) in group II and group III. While there was a slight decrease in group II, significant decrease in group III in the perimeter of the sagittal section of the BEC in group II when compared to the control (Table 2). There was a significant decrease in both the CSA and the perimeter of the sagittal section of the hippocampus in group II and group III when compared to the control (Table 2).

**Table 2:** Indicating the CSA of the brain excluding the cerebellum, its perimeter, CSA of the HC and its perimeter in different groups.

Group	Wt	Brain—cerebellum		Cerebellum		Whole brain	Hippocampus	
		Area	Perimeter	Area	Perimeter	Area	Area	Perimeter
Group I Saline	2	110.80 ± 2.55	54.38 ± 1.65	16.16 ± 0.21	16.35 ± 0.31	126.96	5.63 ± 0.09	9.03 ± 0.10
Group II Sco 6 mg/kg/d—21 d	1.66	81.77 ± 0.56**	49.84 ± 0.14	12.26 ± 0.14	13.07 ± 0.11	94.03	3.45 ± 0.05**	7.40 ± 0.10**
Group III Sco 4 mg/kg/d—28 d	1.44	78.82 ± 1.41**	46.93 ± 0.71**	11.25 ± 1.82	13.17 ± 1.25	90.07	1.92 ± 0.21**	5.95 ± 0.20**
Group IV Iva 5 mg/kg/d—15 d	2	103.80 ± 0.29^##^	52.24 ± 1.06^#^	16.07 ± 0.08	15.84 ± 0.39	119.87	3.57 ± 0.03^##^	7.07 ± 0.03^##^
Group V Iva 10 mg/kg/d—15 d	2.11	112.33 ± 1.79^##^	52.40 ± 1.12^##^	16.62 ± 0.36	16.76 ± 0.58	128.95	3.97 ± 0.19^##^	7.50 ± 0.20^##^
Group VI donepezil 0.5 mg/kg/d—15 d	2.19	109.80 ± 3.10^##^	54.57 ± 1.11^##^	15.57 ± 0.56	16.53 ± 0.64	125.37	3.75 ± 0.05^##^	7.30 ± 0.10^##^

Values are expressed as mean ± SEM of (8–10) rats. Analyzed using by one way ANOVA p < 0.01. **Highly significant (P > 0.01) difference from control group. ^#^Significant (P > 0.05) and ^##^highly significant (P > 0.01) difference from dementia control group.

#### Histopathological results

In the present study, we investigated the histopathological changes to record the changes in situ. We recorded some parameters as the cellular density, the organization, and the thickness and number of cellular rows of the pyramidal layers in the CA and granular cells of the DG which could display the level of neurodegeneration^20^. We also investigated the histopathological characteristic of each neuronal cell type of the hippocampus.

#### The pyramidal layers

**Figure 3:**
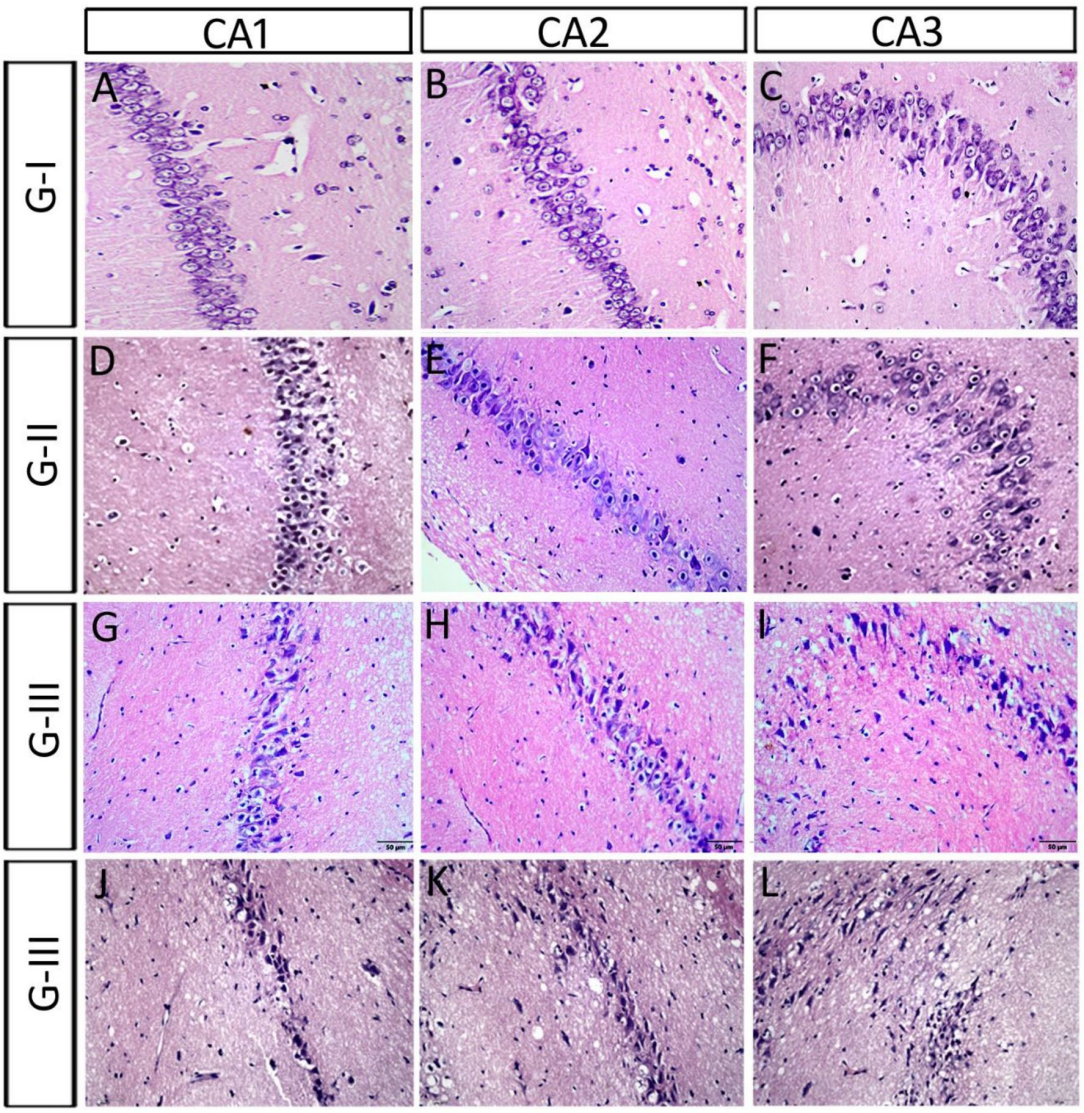
showing the microscopic features of scopolamine administrated groups: (**A**–**C**) the pyramidal layers of the control group I. (**D**–**F**) In group II there were signs of mild neurodegeneration, such as, Balloon cells (BC) with a remnant of the cytoplasm encircling the balloon. (**G**–**I**) In group III, there were more signs of neurotoxicity after the ES, for example complete ballooning of the entire cells (balloon achromatic cells) in all hippocampal subfields. (**J**–**L**) One rat of the ES group (III) showed a highly deformed hippocampus. CA1-CA2 pyramidal cells had been deformed with dysmorphic shapes and CA3 disappeared and few vacuoles were seen instead.

In the control group, the cells were arranged closely with consistent size. They showed a well-defined cytoplasm, cytoplasmic and nuclear membrane, and clearly visible nucleoli (Table 3; Fig. 3A–C). In Group II, the thickness of the pyramidal layer (the distance between the 2 imaginary lines passing over and below the pyramidal cells) decreased significantly in CA1, and insignificantly in CA2 and CA3, when compared to those of the control (Table 3; Fig. 3D–F). The exposure to Sco resulted in signs of mild neurodegeneration, such as, some Balloon cells (BC). We noticed that this phenomenon in group II by the appearance of the nucleus as a condensed densely eosinophilic core, meanwhile, the cytoplasm is present and appeared foamy. In Group III, the thickness of the pyramidal layer decreased significantly in CA2 and CA3, when compared to those of the control (Table 3; Fig. 3G–I). Moreover, the CA3 and hilum showed some elongated cells, along with large distances between the cells, which is the main cause of the increased thickness of this layer (Fig. 3I). There were elongated cells in the pyramidal layer of all subfields of cornu ammonis (CA1, CA2 and CA3) (Fig. 3G–I). There were exaggerated signs of neurotoxicity in one rat after the ES (Fig. 3J–L). CA1 pyramidal cells were dramatically reduced, arranged in one-two rows and became intensively stained (Fig. 3J). Moreover, CA2 pyramidal cells had been deformed with dysmorphic shapes such as triangular, elongated, star shape and some balloon cells with cytoplasmic achromasia (Fig. 3K). Large spaces and many nuclei of glia cells had been seen between CA2 cells (Fig. 3K). Most of CA3 cells disappeared, and few vacuoles were seen instead (Fig. 3L).

**Table 3:** Indicating the SP-thickness, number of rows of the pyramidal layer of various HC subfields (CA1, CA2 and CA3), SG-thickness of the granular layer of DG, number of rows and number of cells in the hilum in different groups. *Sco* scopolamine, *Iva* ivabradine, *HC* hippocampus, *CA* cornu ammonis, *DG* dentate gyrus, *H* hilum, *SG* stratum granule.

G	Treatment	CA1		CA2		CA3		DG		H.
		Thickness	Rows	Thickness	Rows	Thickness	Rows	Thickness	Rows	^#^
I	Saline	77.16 ± 3.34	3	92.20 ± 2.38	3	102.26 ± 0.94	3	90.56 ± 3.86	5.0	25
II	Sco 6 mg/kg/d for 21 d	53.68 ± 0.48**	3	83.17 ± 2.85	3	98.47 ± 3.01	3	64.67 ± 2.75**	5.0	28
III	Sco 4 mg/kg/d for 28 d	69.35 ± 0.35	3	66.25 ± 0.55**	2	38.85 ± 0.95**	1	53.62 ± 1.68**	4.0	8
IV	Sco 4 mg/kg/d for 28 d Iva 5 mg/kg/d for 15 d	69.83 ± 1.80	2, 3, 4	74.73 ± 1.40	2, 3	46.77 ± 3.19	1, 2	81.20 ± 3.50^##^	5.0	23
V	Sco 4 mg/kg/d for 28 d Iva 10 mg/kg/d for 15 d	76.18 ± 3.39	3	70.32 ± 1.82	2, 4	89.72 ± 6.64^##^	2	85.83 ± 3.04^##^	5, 7	24
VI	Sco 4 mg/kg/d for 28 d donepezil 0.5 mg/kg/d for 15 d	84.48 ± 0.60^#^	3, 4	77.27 ± 3.08	2, 3	86.23 ± 2.97^##^	1, 3	89.37 ± 0.24^##^	5, 7	14

Values are expressed as mean ± SEM of (8–10) rats. Analyzed using by one way ANOVA P < 0.01. **Highly significant (P > 0.01) difference from control group. ^#^Significant (P > 0.01) and ^##^highly significant (P > 0.01) difference from dementia control group.

#### The dentate gyrus (DG) and the hilum

**Figure 4:**
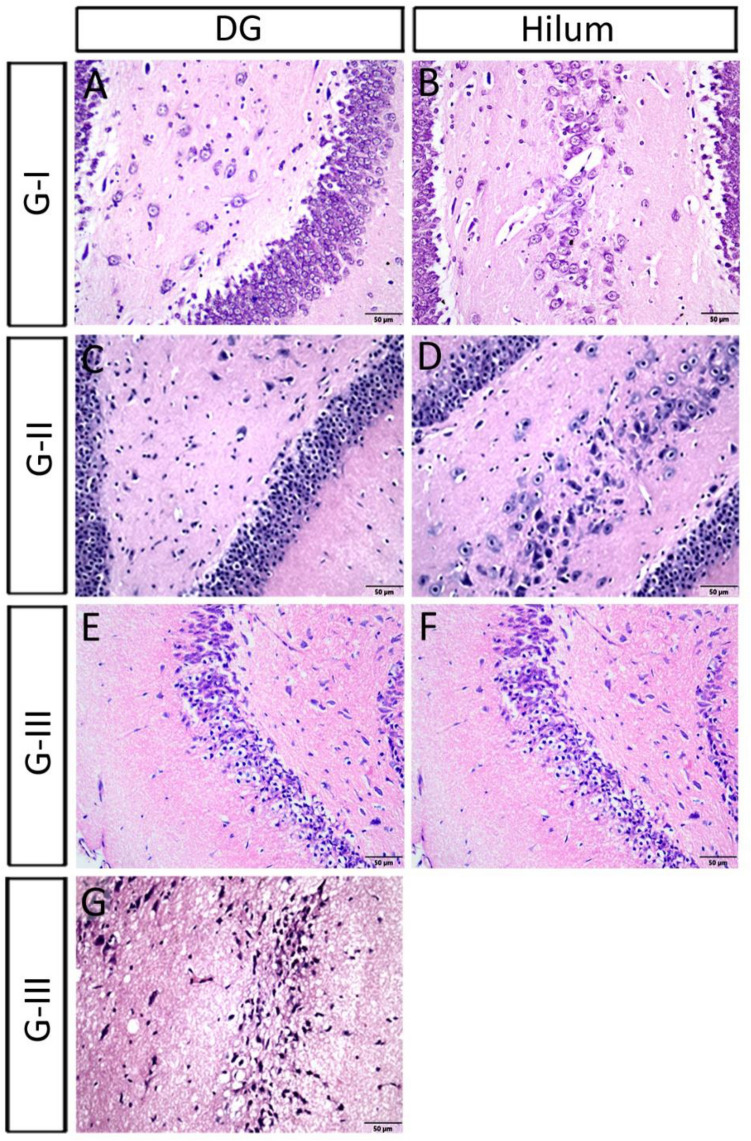
Showing the microscopic features of scopolamine administrated groups: (**A**,**B**) the granular layer and cells in the hilum of the control. (**C**,**D**) In group II, most of the granular layers and hilum cells became BC cells. (**E**,**F**) In group III, both densely stained elongated cells and a lot of BC were shown in the granular layer and the cells of the hilum. (**G**) A rat of the ES group showed a severely deformed hippocampus, where the DG had no hilum and the granular cells were severely reduced, with only a few remaining condensed, and dysmorphic cells and separated by large spaces.

In the control group, the granular cells of DG appeared rounded with large, rounded nuclei contained prominent nucleoli and were surrounded with thin cytoplasm. It was noticed a few small, dark eosinophilic cells variable in shape towards the interior of the DG. There was normal density of glial cell nuclei (Table 3; Fig. 4A,B). In group II and group III, there was a significant decrease in the thickness of the granular layer of DG when compared to that of the control (Table 3; Fig. 4C–F). In group II, the majority of cells in the granular layers and the hilum were BC. We noticed also a moderate density of glial cell nuclei which indicates a mild gliosis. In group III, SG of the DG, along with the cells of the hilum, involved both densely stained elongated cells and a lot of BC. There was moderate density of glial cell nuclei which indicates a mild gliosis. One rat of the ES group (III) showed a highly deformed hippocampus. Its DG had no hilum and the granular cells had been drastically reduced leaving a few remaining cells. The remaining cells became condensed, dysmorphic, and separated by large spaces (Fig. 4G).

Taken together, the behavioural test, ELISA analysis and histopathological results showed that the extended scopolamine administration group showed the greatest signs of behavioural impairment, learning and memory deficit and demented brain and degenerated hippocampus. Therefore, rats exposed to ES were used for further analysis of the Ivabradine effect.

#### Effects of the Iva administration

We used three groups of ES rats (Sco 4 mg/kg/day for 28 days) for further analysis, after cessation of Sco administration. Group IV, which received low dose of Iva 5 mg/kg/day for 14 days; group V, which received high dose of Iva 10 mg/kg/day for 14 days; and group VI, which received Donepezil, one of the already validated medication for Alzheimer disease (0.5 mg/kg/day for 14 days).

#### Histopathological and immunological results

##### The brain size

In the three groups, the brain weight was restored in group IV, V and VI to the healthier average, when compared to group III (Table 2). This was confirmed by the significant increase in CSA and perimeter of the sagittal section of the BEC, when compared to those of group III. Furthermore, there was a significant increase in the CSA and perimeter of the sagittal section of the hippocampus when compared to those of group III (Table 2).

##### The pyramidal layers

**Figure 5:**
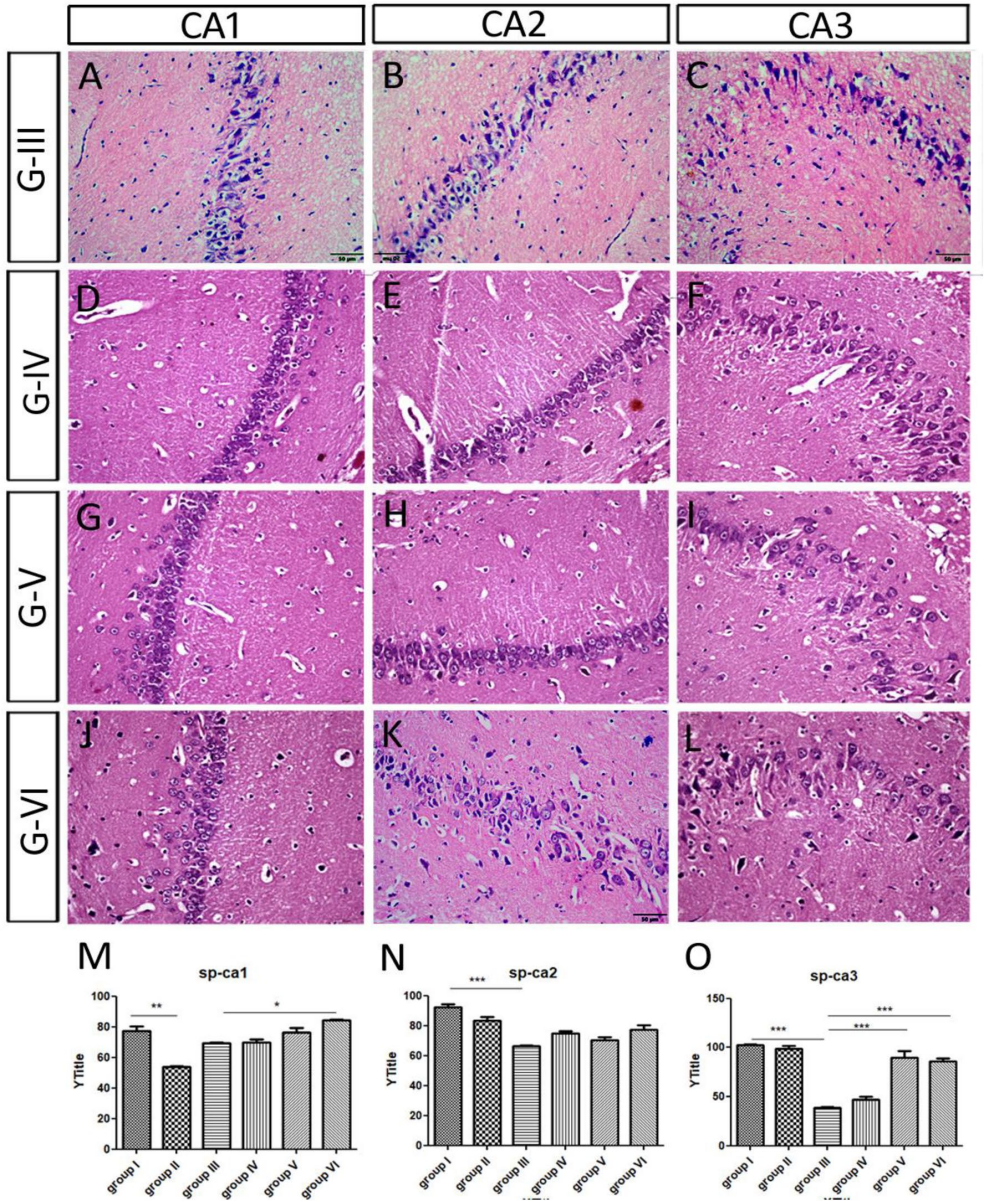
Showing pyramidal layers of the low and high dose ivabradine and donepezil treated groups. More normal healthy cells and less deformed cells were seen in the pyramidal layer of the three groups. (**A**–**C**) The pyramidal layer of ES group III. (**D**–**F**) In group IV, low ivabradine dose, the thickness and the number of the pyramidal layers in CA1, CA2 and CA3 were increased. (**G**–**I**) In group V, high ivabradine dose, the thickness of the pyramidal layer increase in CA1, CA3 and CA3 and there were significant distances between cells. (**J**–**L**) In group VI, donepezil, the shape was comparable to that of the high ivabradine dose. Additionally, the pyramidal layer of CA2 and CA3 showed few densely stained cells. (**M**–**O**) Quantification of the thickness of pyramidal layers in all groups of the experiment including the control (I), the scopolamine (II), the ES (III), the low ivabradine (IV) and high dose (V)of ivabradine and donepezil (VI) on of ES administrative group.

The pyramidal layer of CA1, CA2, CA3 and hilum in the three groups (IV-VI) showed more normal healthy cells and less deformed cells than group III (Fig. 5A–L). In Group IV, the thickness and the number of the pyramidal layers in CA1, CA2 and CA3 were increased, however insignificantly, when compared to those of group III (Table 3; Fig. 5D–F,M–O). Notably, the pyramidal layer of CA2 showed few dense cells and were separated by large distances (Fig. 5E). In Group V, the thickness of the pyramidal layer restored significantly in CA3, along with insignificant increase in CA1 and CA2, when compared to those of group III (Table 3; Fig. 5G–I,M–O). Notably, most pyramidal cells in CA2 and CA3 were separated by large distances in between (Fig. 5H,I). In Group VI, the thickness and the number of the pyramidal layers in both CA1 and CA3 increased significantly, and insignificantly in CA2, when compared to those of group III (Table 3; Fig. 5J–L,M–O). The pyramidal layer of CA2 and CA3 showed few densely stained cells (Fig. 5K,L). Additionally, most pyramidal cells in CA3 were separated by large distances in between, resulting in increasing its thickness (Fig. 5L).

Moreover, the severely affected rat exhibited DG with no hilum and the granular cells had been drastically reduced and became condensed, irregular and surrounded by large space. CA3 disappeared and few vacuoles were seen instead. CA2 pyramidal cells had been deformed with irregular shapes. Moreover, large space and many nuclei of glia cells had been seen in between CA2 cells. CA1 pyramidal cells were dramatically decreased and arranged in one row and became intensively stained.

#### The dentate gyrus and the hilum

**Figure 6:**
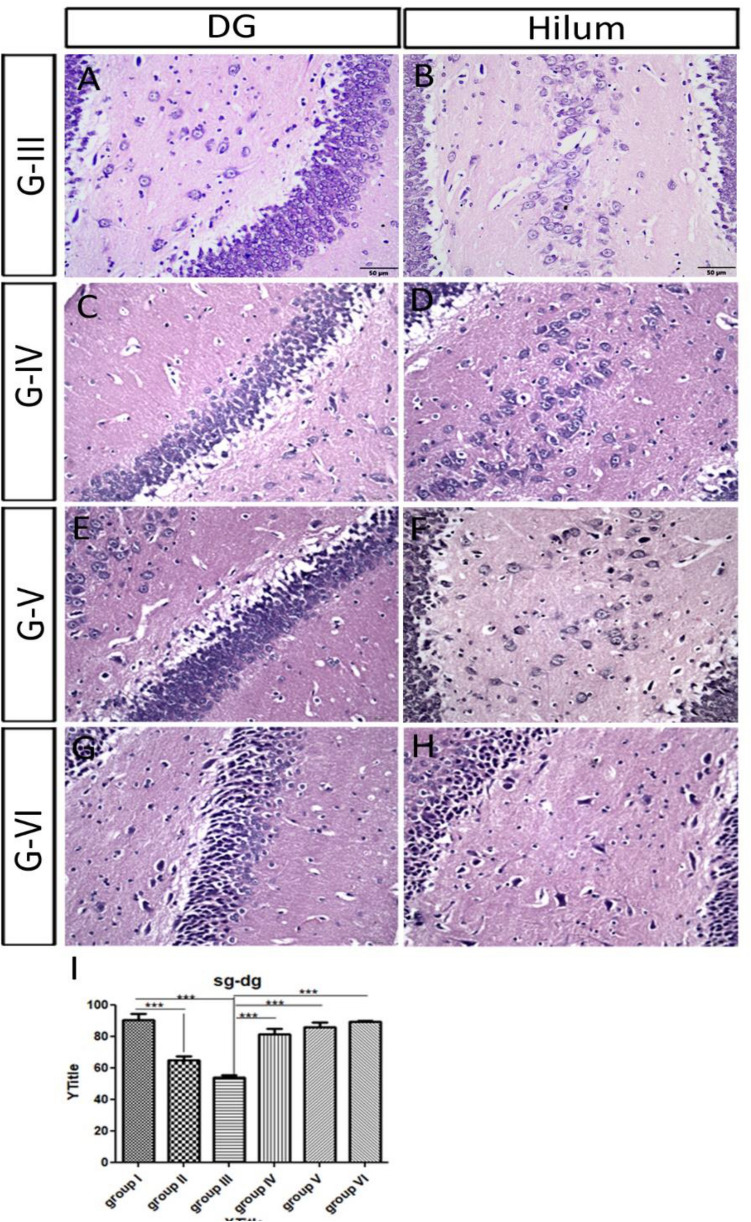
Showing the dentate gyrus of low and high dose ivabradine and donepezil treated groups. (**A**,**B**) The cells degenerated in the granular layer and in the hilum of the ES group III. (**C**,**D**) Group IV, low ivabradine dose. (**E**,**F**) Group V, high ivabradine dose, (**G**,**H**) group VI, donepezil. In the later three groups, the mean number of the cells per section in the hilum increased. The inner layers of the granular layer were dense and small. (**M**–**O**) Quantification of the thickness of granular layer of the dentate gyrus in all groups of the experiment including the control (I), the scopolamine (II), the ES (III), the low ivabradine (IV) and high dose (V) of ivabradine and donepezil (VI) on of ES administrative group.

In the three groups, there was a significant increase in the thickness of the granular layer of the DG (Table 3; Fig. 6A,C,E,G,I). The mean number of cells in the hilum increased when compared to those of group III (8 cells) (Table 3; Fig. 6B,D,F,H). The inner layers of the granular layer in DG were dense and small in the three groups. There were small distances remained between the cells of the outer most layer in group IV (Fig. 6C), which has been recovered in the ES group and donepezil group and showed outer normal cells in the granular layer of DG. There was high density of glial cell nuclei which indicating active gliosis in the three groups. The cells of the hilum were triangular and densely stained after donepezil administration (Fig. 6H).

### Congo red staining

**Figure 7:**
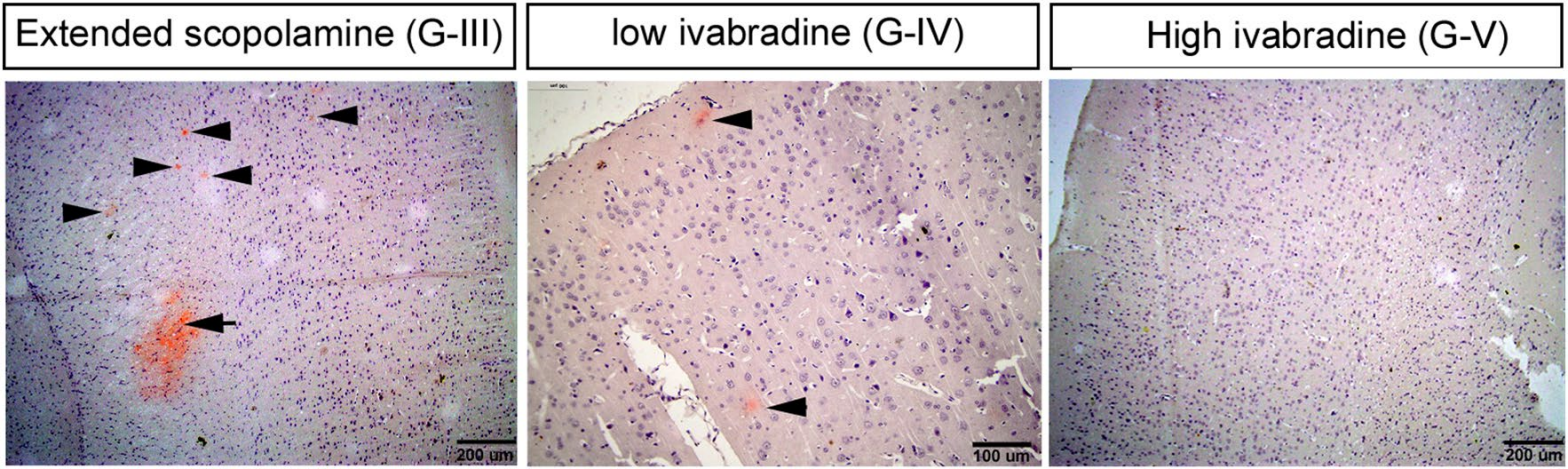
Showing the amyloid plaques stained with congo red. On the left panel, there are many plaques of both large and small sizes in the extended scopolamine group. In the middle, after low ivabradine the plaques become less frequent and of small sizes only. The plaques disappear from the cortex and hippocampus in case of ES group.

The cut hemispheres were stained with Congo red dye to detect the deposition of beta amyloid plaques^21^. After administration of Sco in group II, merely one plaque (diameter 30 µm) was observed compared to the control group which has no plaque. Whereas ES in group III, resulted in multifocal deposition of amyloid beta plaques (8 plaques) with variable sizes (20 × 3, 30, 50, 60, 70 and 100 µm) were observed (Fig. 7). These results confirmed that the ES has the highest adverse effects on the brain.

After cessation of Sco and administration of low dose of Iva in G-IV, the deposition of amyloid beta plaques did not disappear completely, however, they decreased in number (5 plaques) and reduced in size (from 20 to 40 µm) when compared to those in G-III. Interestingly, in case of administration of higher dose of Iva in group V for the same period, the deposition of amyloid beta plaques disappeared in all stained slides. The donepezil administrated group showed comparable results to those of the higher dose of Iva, where the deposition of amyloid beta plaques disappeared.

### HCN1 expression

**Figure 8:**
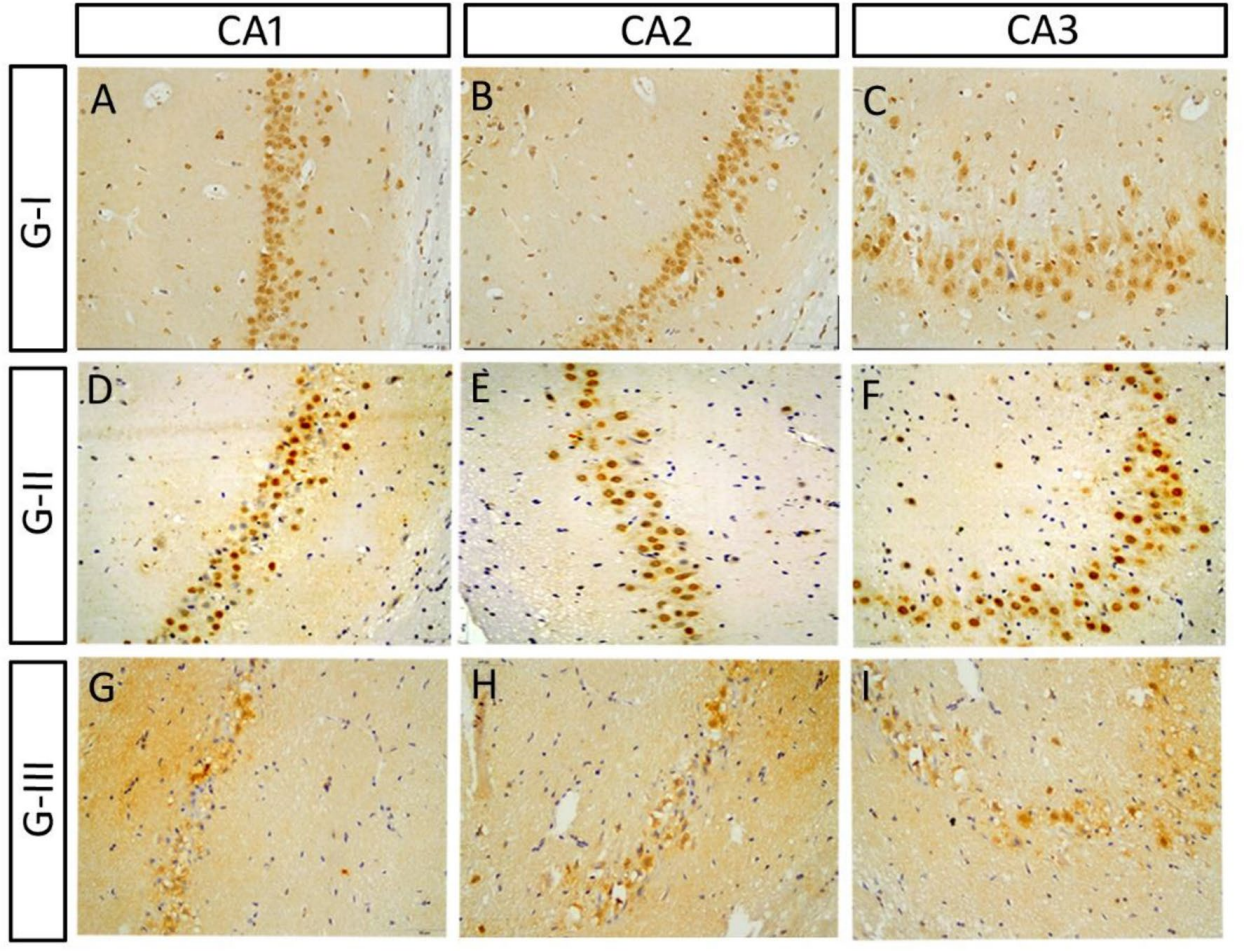
Expression of HCN in scopolamine administrated groups in CA: (**A**–**C**) The HCN1 immunoreactivity in the pyramidal layer of CA of the control group. (**D**–**F**) The HCN1 immunoreactivity in group II was comparable to that of the control group in CA. (**G**–**I**) The HCN1 immunoreactivity decreased significantly in ES group III in CA.

**Figure 9:**
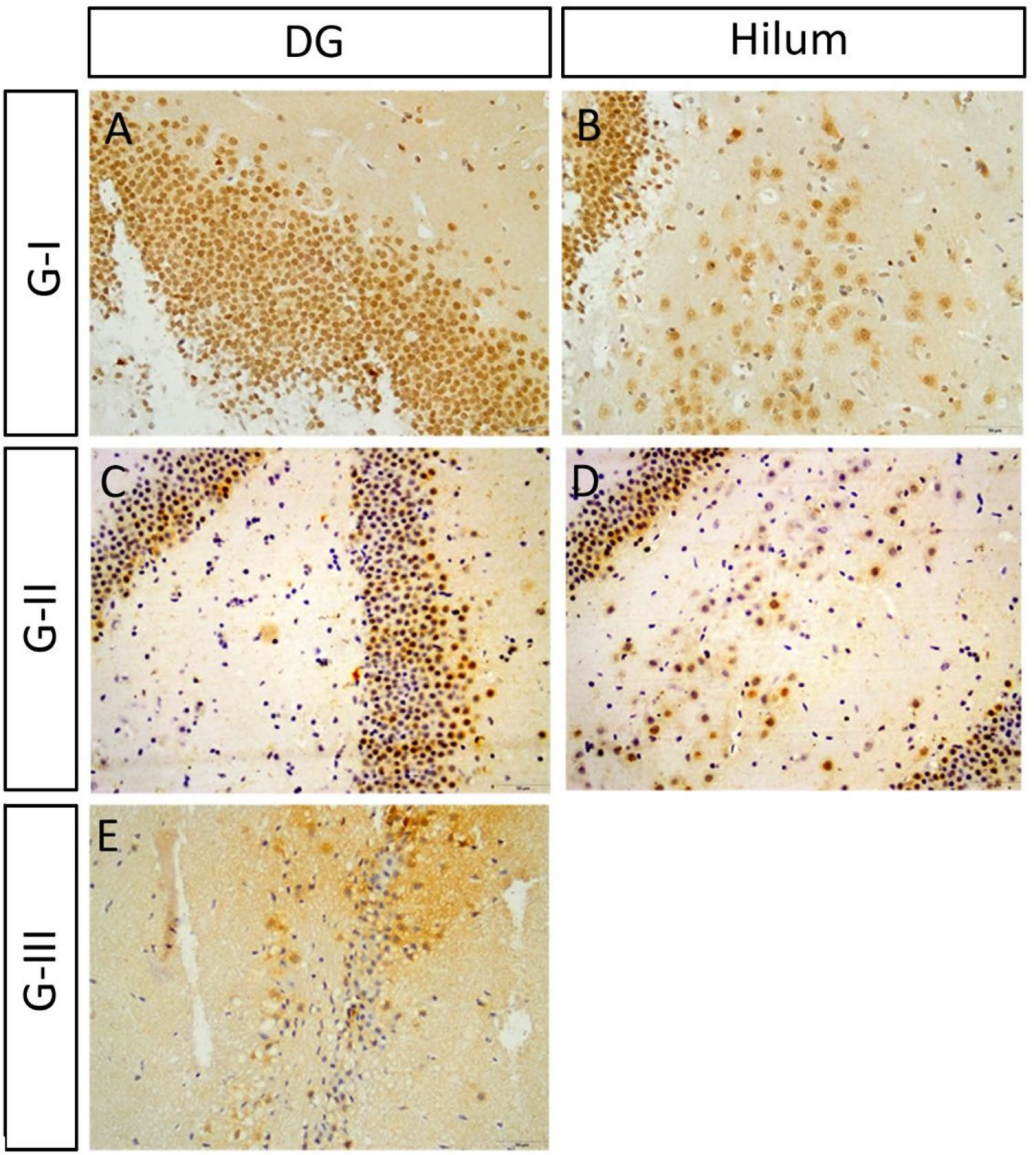
The expression of HCN1 in scopolamine administrated groups expression in DG and hilum: (**A**,**B**) The HCN1 immunoreactivity of the control. (**C**,**D**) The HCN1 immunoreactivity in group II was comparable to that of the control group in DG and hilum. (**E**) The HCN1 expression decreased significantly after ES in group III in DG and hilum.

**Figure 10:**
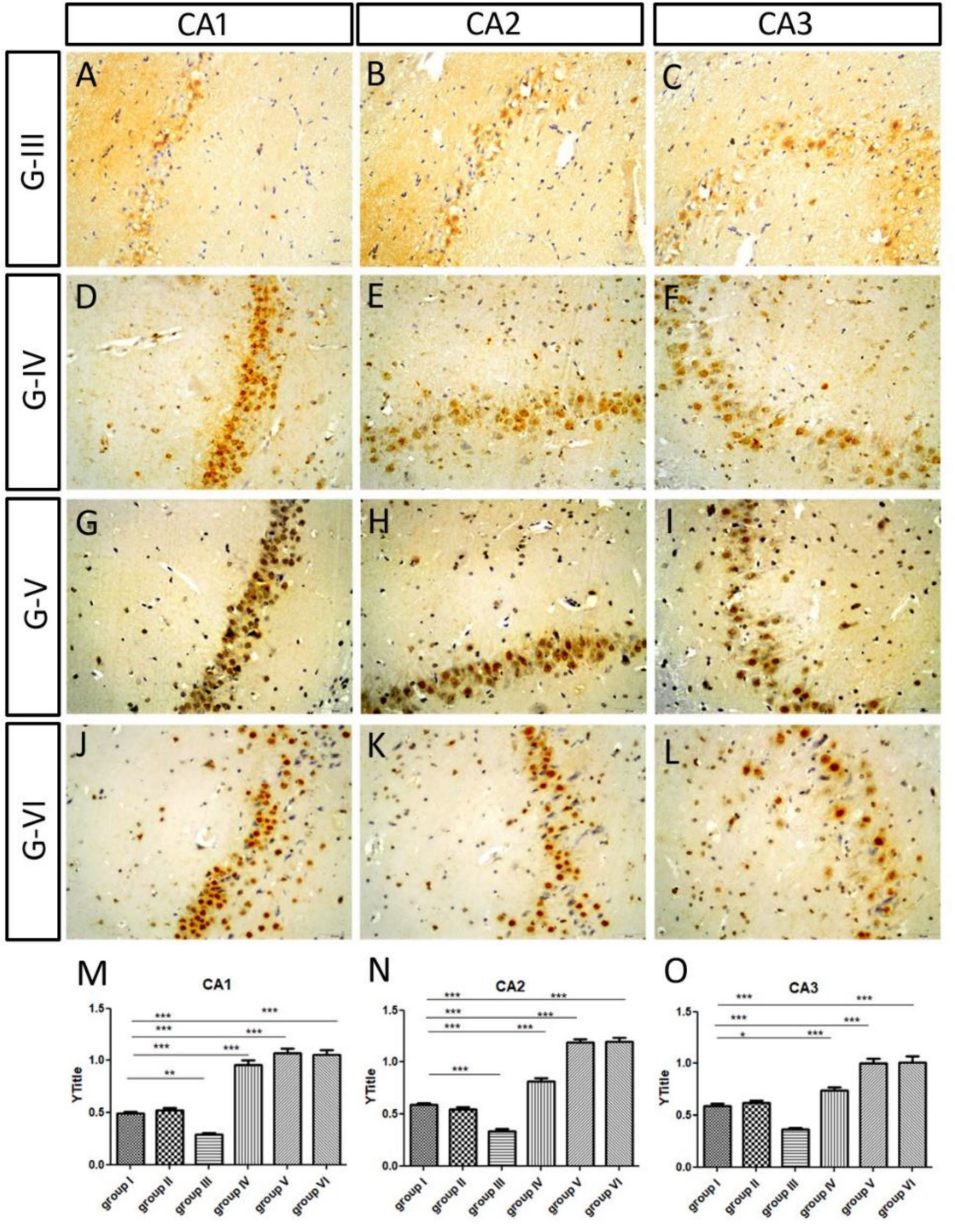
The expression of HCN in low and high doses of ivabradine and donepezil groups in CA: (**A**–**C**) The HCN1 immunoreactivity of the ES group III in CA. The HCN1 expression in CA increased in group IV, low ivabradine dose (**D**–**F**), increased dramatically in group V, high ivabradine dose (**G**–**I**). In group VI, donepezil (**J**–**L**), was comparable to that of the high ivabradine treated group. (**M**–**O**) Quantification of the optical density of HCN1 immunoreactivity in CA1 (**M**), in CA2 (**N**) and in CA3 (**O**) in different groups.

**Figure 11:**
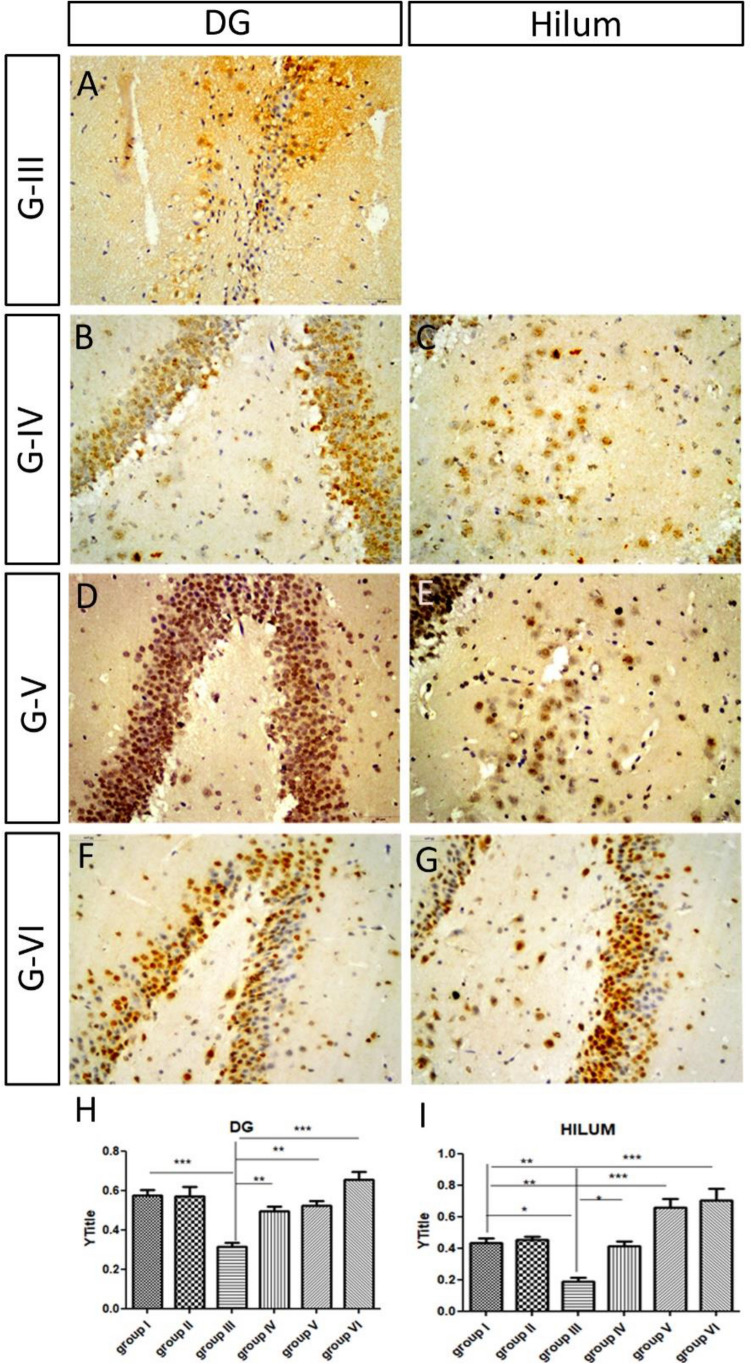
The expression of HCN in low and high doses of ivabradine and donepezil groups in DG and hilum: the high ivabradine dose and donepezil-treated rats showed a comparable increase in HCN1 immunoreactivity, which was much higher than the control group. Note that the hilum disappeared in the same group. (**H**,**I**) Quantification of the optical density of HCN1 immunoreactivity in DG (**H**) and in the cells of the hilum (**I**) in different groups.

All hippocampal sections were processed simultaneously, and images were captured using fixed microscope parameters. Briefly, at least 10 different HCN1-immunoreactive cells in different subfields of the hippocampus from 3 different sections of the three replicates were measured. The mean and maximum grey intensity were measured using Image J software and the optical density of HCN1 immunoreactivity was calculated according to the following equation: OD = log (max gray intensity/mean gray intensity). We found that the OD of HCN1 immunoreactivity in group II, after 3 weeks of Sco injection, was comparable to that of the control group in different subfields of the HC (Table 4; Figs. 8A–F and 9A–D). However, it decreased significantly after ES in group III in different subfields of the hippocampus when compared to the control and group II (Table 4; Figs. 8G–I and 9E). After application of Iva, we observed that the optical density of HCN1 immunoreactivity increased dramatically in low and high Iva group IV, V and donepezil group VI in all hippocampal subfields when compared to the ES group III (Table 4; Figs. 10A–O, 11A–I).

**Table 4:** Summarizing the optical density of HCN1 immunoreactivity in various HC subfields (CA1, CA2, CA3, DG, and hilum) in different groups:

G	Treatment	CA1	CA2	CA3	DG	Hilum
G- I	Saline	0.49 ± 0.02	0.59 ± 0.01	0.59 ± 0.02	0.57 ± 0.03	0.43 ± 0.03
G- II	Sco 6 mg/kg/d for 21 d	0.52 ± 0.02	0.54 ± 0.03	0.62 ± 0.02	0.57 ± 0.05	0.45 ± 0.02
G- III	Sco 4 mg/kg/d for 28 d	0.29 ± 0.02**	0.34 ± 0.02**	0.36 ± 0.02**	0.32 ± 0.02**	0.19 ± 0.02*
G- IV	Sco 4 mg/kg/d for 28 d followed by Iva 5 mg/kg/d for 15 d	0.96 ± 0.05**^##^	0.81 ± 0.03**^##^	0.74 ± 0.04*^##^	0.50 ± 0.02^##^	0.42 ± 0.03^#^
G- V	Sco 4 mg/kg/d for 28 d followed by Iva 10 mg/kg/d for 15 d	1.07 ± 0.05**^##^	1.19 ± 0.03**^##^	1.00 ± 0.05**^##^	0.52 ± 0.03^##^	0.66 ± 0.05**^##^
G- VI	Sco 4 mg/kg/d for 28 d followed by donepezil 0.5 mg/kg/d for 15 d	1.06 ± 0.04**^##^	1.20 ± 0.04**^##^	1.01 ± 0.06**^##^	0.65 ± 0.04^##^	0.70 ± 0.08**^##^

Values are expressed as mean ± SEM of (8–10) rats. Analyzed using by one way ANOVA p < 0.01. *Significant (P > 0.05) and **highly significant (P > 0.01) difference from control group. ^#^Significant (P > 0.05) and ^##^highly significant (P > 0.01) difference from dementia control group.

We used scopolamine to induce dementia model (4 mg/kg for 28 days), then we investigated the effect of ivabradine (for the consecutive 15 days) on cognitive functions. To ensure that the amelioration was due to either Iva or Donepezil, there was a group administrated scopolamine (4 mg/kg and 28 days) and left for 15 days without administrating any medication. The behavioural tests were performed then sacrificed, biochemical and the conventional histopathological examination were assessed. We found that this group did not recover to the level of the control group (not published data).

## Discussion

In this study, the therapeutic effects of Iva, a known antianginal and antiarrhythmic agent, had been investigated and compared to that of donepezil, the already validated medication for AD. Three behavioural tests were used for evaluating amnesia (spatial learning and memory function^18^); the passive avoidance, Morris water maze, and novel object recognition. Our study showed that the injection of Sco has impaired the ability of learning and memory. This was detected by behavioural tests and confirmed by ELISA and histopathology.

By comparing the weight of the brain, the CSA and the perimeter of both BEC and the hippocampus of the Sco administrated group to the control, there was a decrease in these parameters which became more marked in ES group. A similar effect has been reported after chronic alcohol abuse^22^, where cognitive impairments were associated with neurodegeneration and volume loss in the human hippocampus^23^.

All main neurodegenerative diseases are linked to oxidative stress, which has long been considered as a possible therapeutic target^24^. Inflammation and oxidative stress occur before the cardinal neuropathological signs of Alzheimer's disease^25^. In the present study, we got similar results to those of Liu and his colleagues who reported that Sco treated mice showed increase content of MDA level and a significant decrease in the content of SOD in memory impaired mice^26^. We also recorded a significant decrease in TAC level in the hippocampus of the ES group. These deleterious effects of Sco had been stopped and there was a significant decrease in MDA levels and significant increase in SOD and TAC levels in low and high Iva groups, as that shown in donepezil treated group, when they compared to ES group.

In the present study, there was a significant increase in the level of proinflammatory cytokines such as TNF**-**α, IL-2 and IL-6 in ES group, when compared to control group. The high levels of cytokines is believed to cause inflammation and cholinergic neuronal degeneration that have a role in the development of the degenerative alterations and cognitive impairment associated with Alzheimer's disease^27^. These high levels of proinflammatory cytokines subsided significantly after treatment with either low or high Iva as well as after donepezil treatment. Zuo and his colleagues confirmed the Iva anti-inflammatory properties, as well as its impact on the expression and release of the proinflammatory cytokines in streptozotocin-induced diabetic cardiomyopathy in mice^28^.

In the present study, the cognitive impairment was associated with reduction of pyramidal neurons numbers and thinning of the pyramidal layer along the entire CA after Sco administration. Similar findings have been reported in the hippocampal CA1 and CA3 regions after global brain ischemia^29^, and in CA1 in the mild or moderate AD patients^30^. Likely, the significant volume loss of DG granular layer after Sco administration is similar to that reported after irradiated hippocampus region of rats’ head with a minimum of (2 ×) doses of 150–200r X‐rays^31^ which has been attributed to impairment of hippocampal neurogenesis^23^.

In the present study, especially after ES, vigorous signs of neurotoxicity appeared histologically, which reached to a severe deformed hippocampus in one rat. The balloon cells (dark pyknotic nuclei with intense vacuolations) in the CA with cortical disorganization are associated also with drug-resistant epilepsy^32^ and, in a recent study, after high doses of caffeinated energy drinks in adult male albino rats^33^. The severely affected rat resembled those described in stroke model of dementia (established by bilateral occlusion of common carotid arteries)^34^**,** in cadmium subcutaneously injected rat^35^ and in oral administration of AlCl3 to rats^36^. In those models, neurons of CA and DG underwent marked distortion, reduced cellular density, loose arrangement, and misalignment, disappeared cell outline, shrunken cytoplasm, obscured nuclear boundary and the nucleolus disappeared. Additionally, some cells shrank and exhibited vacuolated eosinophilic cytoplasm and hyperchromatic pyknotic shrunken nuclei (apoptotic changes) associated with the appearance of pericellular haloes. The aforementioned results revealed that the ES (group III) induced more significant defects of memory accompanied by many histopathological signs of AD validating the success of AD model establishment. Therefore, we continued the evaluation of the Iva administration on ES rats (group III).

Grossly, Iva and donepezil improved the Sco induced atrophy of the brain in group IV, V and VI. There was an increase in the weight of the brain when compared to group III. This could be confirmed by quantification of the CSA and perimeter of the sagittal section of the BEC and the hippocampus, which significantly increased when compared to those of group III (ES). Treatment with Iva and donepezil reduced the degenerative changes and restored the normal hippocampal cytoarchitecture including the cell populations and the regular distribution. Similar effects have been reported for quercetin treatment^35^ and mesenchymal stem cells treatment^36^.

In the current study, the morphometric data revealed restoration of the pyramidal layer thickness of CA1 and CA3 especially after high Iva and donepezil. A similar neuroprotective effect has been reported for Luteolin^37^ and Myricetin^38^ after intracerebroventricular injection of streptozotocin (ICV STZ) and for triazine derivative, after STZ^39^. Likewise, in these models and ours, the number of hippocampal CA3 pyramidal neurons raised and the learning and memory have been improved. Luckily, there was a significant increase in the thickness of the granular layer of the DG after high Iva and after donepezil administration, as that described for topiramate^40^. Similar effects have been reported for melatonin treatment^41^ and for *Centella asiatica* after d-gal and AlCl3 induced neurodegeneration^36^. This might be due to promoting aberrant neuron regeneration and the survival of new-born neurons in the hippocampus^40^.

Sco is an antimuscarinics, which works by blocking the acetylcholine (inhibitor) in the central nervous system (CNS)^42^. It impairs learning and memory through increasing activities of acetylcholinesterase (AChE), butyrylcholinesterase (BuChE), adenosine deaminase (ADA), and lipid peroxidation with a concomitant decrease in levels of nitric oxide (NO), reduced glutathione (GSH), SOD, glutathione S-transferase (GST), and catalase activities^43^. Moreover, Sco increased the expression of inflammatory mediators such as cyclooxygenase2 (COX2), nuclear factor kappa B (NF-kB), TNF-α^44^.

Donepezil can inhibit the activity of BuChE, and adenosine deaminase (ADA), significantly decreases lipid peroxidation and increases levels of NO and antioxidant status^43^. It regulates endocytic trafficking of amyloid precursor protein (APP), via up-regulation of sorting nexin protein-33 SNX33 expression^45^. Our study confirmed its therapeutic effect which lead to restoration of the normal healthy neurons and cytoarchitecture and the HCN1 expression, which indicates a new mechanism for donepezil.

In the present study, the optical density of HCN1 immunoreactivity decreased significantly after ES in group III in different subfields of the hippocampus, indicating the involvement these channels in the Sco-induced neurodegeneration, similar to the effect of chronic cerebral hypoperfusion in rat^46^. Furthermore, the decrease in HCN1 and HCN2 expression in medial temporal lobe epilepsy with hippocampal sclerosis has been suggested to increase the susceptibility to seizures because it reduces the density of I_h_ currents and enhance the excitability of neurons^47^. The pathogenetic mechanism underlies the neuronal discharge activity inconsistency is one of the main discussed hypotheses of the neurodegenerative diseases^48^.

Iva, the broad-spectrum HCN blocker which blocks the HCN-mediated current (I_h_), is effective at reducing seizure susceptibility^14^. Iva, at present, has been approved for use in clinical practice as an adjunct in the treatment of select patients with symptomatic (HFrEF; EF < 40%), as it reduces heart rate by direct action on the sinus node and improve outcomes in HF^49^. Till now, the relationship between HF and AD remains largely unclear. Alzheimer’s disease and HF often occur together and thus increase the cost of care and health resource utilization^50^. A recently proposed risk factor for AD is HF. Decreased cerebral blood flow and neurohormonal activation due to heart failure may contribute to the dysfunction of the neurovascular unit and cause an energy crisis in neurons. This leads to the impaired clearance of amyloid beta and hyperphosphorylation of tau protein, resulting in the formation of amyloid beta plaques and neurofibrillary tangles^51^.

Ion channel dysfunction has been suggested as a potential cause for neurodegenerative diseases^52^. In the hippocampus, neocortex, and cerebellar cortex, HCN1 appears to be the most common isoform present^53^. In the CNS, alteration of the I_h_ current could predispose to the development of neurodegenerative diseases such as Parkinson’s disease^52^. Given the fundamental role played by the HCN channels in the regulation of the discharge activity of neuronal cells, the modulation of their function for therapeutic purposes could be useful in various pathological conditions. Selective inhibition of HCN channels has robust therapeutic scope as anti-convulsants, anti-psychotics, and anti-depressants agents and safe to pulmonary and/or vascular smooth muscle tone^54^. A causal links has been suggested between the lower expression of HCN channels, the occipital alpha rhythm and the reduction of the thalamic alpha rhythm frequency and coherence, which precedes amyloid-β plaque formation in AD^11,55^.

Fortunately, we found that both of Iva and donepezil has improved the Sco-induced memory impairment and cognitive deficits in rats and ameliorated the histopathological alterations. Notably, we also realized, from our data, a significant amelioration in the behavioural and histopathological signs of AD after high dose of Iva which is comparable to those of donepezil. It has been reported that there was possible causal relationships between key markers associated with AD (Beta Amyloid, HCN expression and Acetylcholine hypothesis). i.e., decreased expression of HCN channels is correlated to AD^56^. Moreover, loss of HCN1 surface expression is associated with decreased Ih amplitude and a hyperpolarizing shift in voltage-dependence of activation (gating)^57^. The important link to the levels of expression of Ih and HCN protein have recently been shown to be regulated by neuronal activity^58^. Since the most consistent effect of ACh on hippocampal pyramidal neurons appears to be a pronounced membrane depolarization coupled with increased membrane resistance^59^. Therefore, the resulting lower neuronal activity of pyramidal neurons caused by Scopolamine might be the cause for lower HCN expression, and the restoration of neuronal activity in case of Donepezil might lead to the restoration of HCN expression.

Taken together, our results supported the hypothesis that the dysfunction of HCN1 channels can underlie neuroinflammation and resulting neuronal dysfunction. In addition, donepezil and Iva can markedly ameliorate the Sco-induced neuronal toxicity by modulating HCN1 expression and function. Furthermore, Iva has a potential neurosupportive therapeutic value for the treatment of insulted neurons in dementia and could be a candidate for a new medication to relieve cognitive dysfunctions which might be partly attributed to blocking of HCN1 channels. This adds new benefits for this drug in patients with HF associated with AD which opens a possible new concern for its neuroprotective effect. However, further studies are essential before considering Iva an effective anti-AD drug.

## Materials and methods

### Animals

Adult male Wistar rats weighing 350 ± 20 g were supplied by the animal house facility, Faculty of Medicine, Assiut University and were housed in groups of four rats per cage at a temperature of 22 ± 2 °C on a 12 h light/dark cycle. Food and water were provided ad libitum. The research was conducted in accordance with the internationally accepted principles for Guide for the Care and Use of Laboratory Animals (NIH Publications No 85-23, revised 1985) and approved by Assiut University Institutional Animal Ethics Committee (Protocol No. 17100668, 2019). This animal study was performed in accordance with ARRIVE guidelines.

### Drug administration

**Figure 12:**
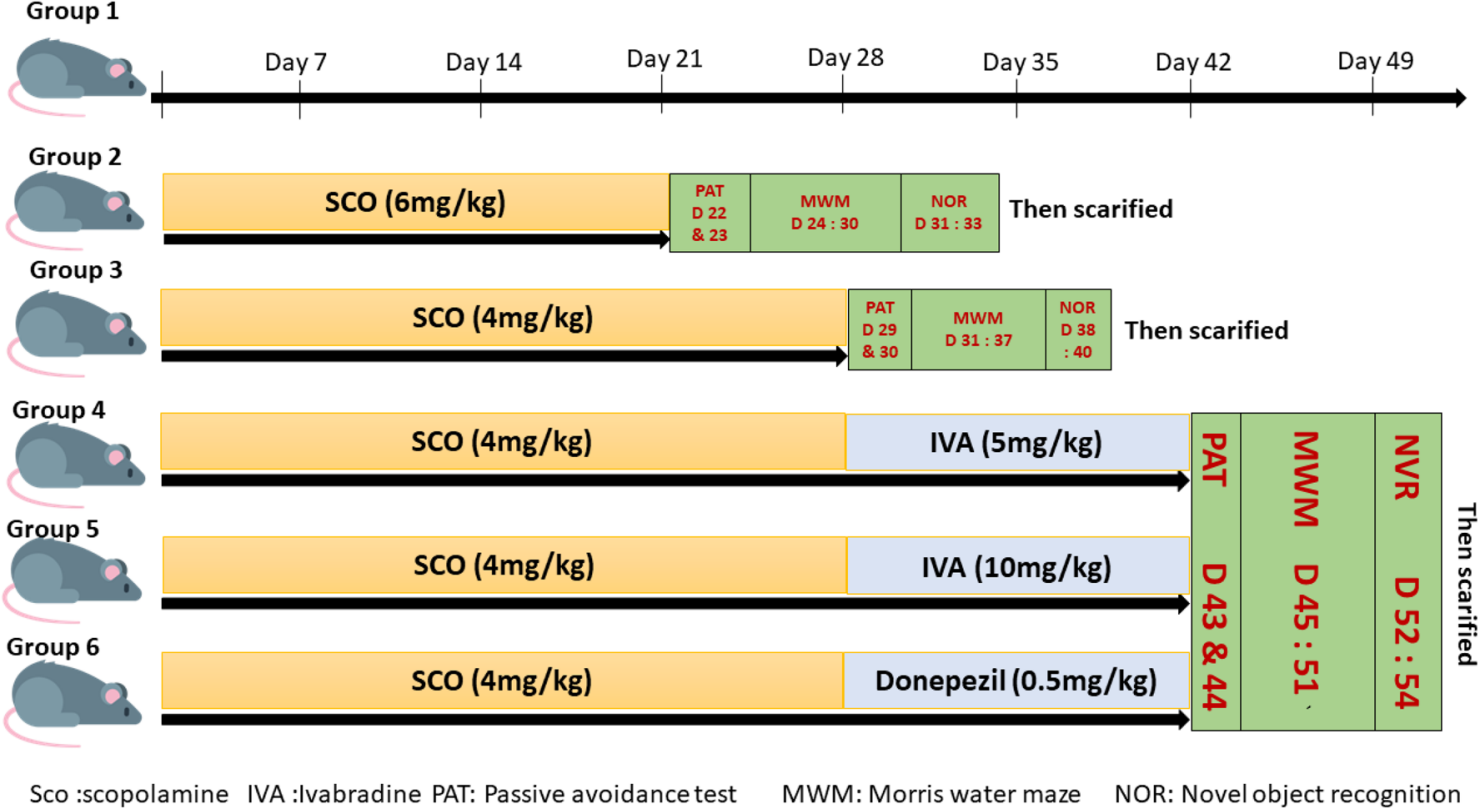
The timeline graphical representation. *Sco* scopolamine, *IVA* ivabradine, *PAT* passive avoidance test, *MWM* Morris water maze, *NOR* novel object recognition.

To analyze the effects of Iva on rat models of Sco induced dementia, animals were randomly divided into 6 groups, each has 8 rats. Sco hydrobromide (Acros Organics, Belgium, 16175), Donepezil (sigma-Aldrich, UK, D6821) and Iva hydrochloride (AK-scientific, USA, E147). First, we validated the Sco induction of dementia. To this end, we used 3 groups; group I the control, which was injected with i.p. saline in equal volumes and regimens to Sco.; group II, Sco-treated group which received i.p Sco, 6 mg/kg/day for 21 days; and group III, Extended Sco-treated (ES) which received i.p Sco for longer period, 4 mg/kg/day for 28 days. Second, we chose the most affecting time/dose protocol to cause obvious demented brain for further analysis of the effect of Iva. To this end, we used 3 other groups after having been administered ES Sco and showed behavioural signs of dementia; group IV, low Iva which received low dose of Iva (5 mg/kg/day) for 2 weeks; group V, high Iva which received high dose of Iva (10 mg/kg/day) for 2 weeks; and group VI, which received donepezil (0.5 mg/kg/day) for 2 weeks. During this study, we analyzed each fifth section for at least three different representative sections. See the timeline graphical representation (Fig. 12).

### Behavioural tests

With the researchers blinded to treatment conditions, behavioural tests were performed as described before; Passive avoidance test^60^, Morris water maze (MWM)^61^ and Novel object recognition test^62^.

### Specimen harvesting

After completion the behavioural tests, overnight fasted animals were anesthetized with thiopental sodium and transcardially perfused by 0.9% cold saline. The whole brain was carefully isolated from each rat and divided into hemispheres. The brain was isolated from each rat and bisected into hemispheres. The left hemispheres were fixed in 10% neutral-buffered-formalin (PFA) for 48 h, followed by washing twice with phosphate buffered saline (PBS). These hemispheres were further processed for paraffine embedding. We analysed each fifth section for at least three different representative rats. The hippocampi of the right hemispheres were immediately dissected on dry ice, wet tissues were blotted dry with a filter paper and stored at − 80 °C to be used for ELISA.

### Biochemical assay

Measurement of the hippocampal level of pro-inflammatory cytokines (tumor necrosis factor-α (TNF- α), interleukin (IL)-2, and IL-6) was performed as described before^63^. Evaluation of hippocampal oxidative stress biomarkers was measured as described before, such lipid peroxidase (malondialdehyde)^64^, superoxide dismutase (SOD)^65^ and total antioxidant capacity (TAC)^66^.

### Histological examination

The paraffine block were cut serially into thin sections (5–6 µm) and the sections were further stained with eosin and haematoxylin for investigating the histological changes. Congo red stain were applied to investigate the amyloid plaques on formalin-fixed, paraffin-embedded tissue sections^21^. A modified Highman's Congo red stain has been used. The technique was performed briefly, paraffine sections were dewaxed in xyline 3 times 5 min each then hydrated in descending grades of ethanol 3 min each. Sections were incubated in congo red solution (100 ml ethanol 50% + 0.6 g congo red) for 40 min, rinsed in distilled water, differentiated quickly in alkaline alcohol solution (1 M NaOH in saturated NaCl solution in 37% ethanol) and rinsed in tap water for 1 min. Sections were counterstain with hematoxylin for 20 s, rinsed for 2 min. Sections were dehydrated in ethanol, cleared in xylene, and finally covered using DPX mounting medium. The amyloid deposits were stained red, and the nuclei were stained blue (Supplementary Information).

Immunohistochemical analysis was also conducted against HCN1 antibody, (diluted 1:50, Cat. No. abx214762 abbexa, UK), to investigate the impact of Iva treatments on the Sco-induced demented brain. The techniques was performed as described by^67^ however the secondary antibody was Ultra Tek HRP Anti-polyvalent kit (Goat anti-mouse, rat, rabbit and guinea pig IgG) was purchased from ScyTek (USA).

### Statistical analysis

Data are expressed as the mean ± standard error of mean (SEM). Statistical analysis was performed by a one-way and two way analysis of variance (ANOVA), using GraphPad Prism 5.03 (GraphPad Software, Inc.). For all statistical comparisons, a P-value of < 0.05 was considered statistically significant.

## Supplementary Information

Supplementary Information.

## Abbreviations

PTZ: Pentylenetetrazole
PICRO: Picrotoxin
PFC: Prefrontal cortex
ST: Striatum
MDA: Malondialdehyde
GSH: Glutathione
AChE: Acetylcholinesterase
ES: Extended scopolamine
IL: Initial latency
STL: Step through latency
TNF-α: Tumor necrosis factor alpha I
L-2 and IL-6: Interleukin2 and interleukin-6
SOD: Superoxide dismutase
TAC: Total antioxidant capacity
CSA: Cross-sectional area
BEC: Brain excluding cerebellum
CA: Cornu ammonis
DG: Dentate gyrus
