# Supplementary material for: Effect of ivabradine on cognitive functions of rats with scopolamine-induced dementia
Source: Sci Rep. 2022 Oct 10;12:16970. doi: 10.1038/s41598-022-20963-5 (PMC9551060; doi:10.1038/s41598-022-20963-5)
Supplement: Supplementary file 1 — Supplementary Information. [file 41598_2022_20963_MOESM1_ESM.doc]

## **Supplementary file**

## Effect of Ivabradine on Cognitive Functions of Rats with Scopolamine - induced Dementia

## Behavioral Tests

- **Passive avoidance task**

The passive avoidance device (Ugo Basile, Italy) consists of an electric grid floor and is separated by a partition with a sliding door into two equal-sized compartments (light and dark). Rat success relies on their inherent preference for darkness. When each rat was inserted into the light compartment, the learning trial began 2 minutes after habituation in the dark compartment. A 2-s period electric foot-shock (1.5 mA) was transmitted through the grid floor as the rat crossed to the dark compartment. The time taken to reach the dark chamber was reported as the initial latency in the acquisition trial (IL). The retention test was carried out 24 hours after the acquisition, where rats were placed in the light compartment again, and the step-through latency (STL) to enter the dark chamber was registered, with a cut-off time of 300 s^1^.

### Morris water maze (MWM)

To assess spatial learning and memory in different groups, the MWM test was performed. In a 1.4-m diameter circular pool filled with opaque water and randomly divided into four quadrants, animals were permitted to swim freely. The test consisted of 6 successive training days (3 tests per day) and a test session (test trial) on day 7. Rats were needed to identify the location of the escape platform submerged 1 cm below the water surface during the acquisition phase and held at the centre of one of the quadrants of the pool. Each rat was permitted to search for a maximum of 90 s for the hidden platform, and those who failed to locate the platform were gently guided and put for 10 s on the platform. The escape latency to the platform was assessed during each training session. Animals got a probe test (retention test) on the 7th day, without the platform. The latency to identify where the platform was previously submerged during the test period (60 s) and the time spent on the target quadrant was calculated^2^.

### Novel object recognition test

The test for novel object recognition was carried out in accordance with the procedures previously described^3^. The assignment was performed in a box with an open field (60 x 60 x 40 cm). First, all animals underwent a habituation session in which they were allowed to freely explore the object-free open-field for 5 min. 24 hours later, the rats were subjected to a sample phase session in which two identical objects (toys) A and B were placed in the field at symmetrical positions approximately 10 cm away from the wall for 5 min. Exploration is defined as the animal looking at, sniffing, or touching it with its head within 2 cm of the object. 24 h following this, the rats underwent a test phase session where they could explore the open field for 5 min with one familiar and one novel object. There were similar textures and sizes to all the objects presented, but distinctive shapes. After each trial, the objects were washed with 10 percent ethanol to remove smells or residues. The exploration times were recorded.


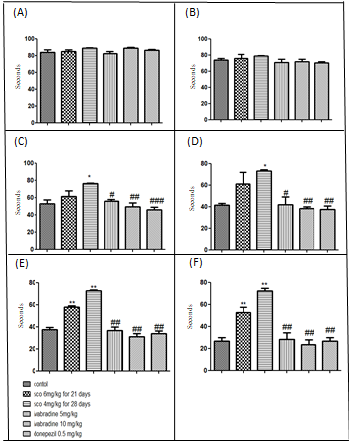


Fig 1 supplementary Effect of ivabradine on escape latency of the acquisition trials in morris water maze in scopolamine induced dementia (in seconds).(A) During 1 ^st^ day trial of the acquisition trials.(B) During 2^nd^ day trial of the acquisition trials.(C) During 3^rd^ day trial of the acquisition trials.(D) During 4^th^ day trial of the acquisition trials.(E) During 5^th^ day trial of the acquisition trials.(f) During 6^th^ day trial of the acquisition trials.

**Biochemical assay**

Measurement of the hippocampal level of pro-inflammatory
cytokines: This experiment was carried out to evaluate the role of pro-inflammatory cytokines in the impairment of cognitive function in scopolamine treated rats and to assess the effect of treatment on the hippocampal level of tumor necrosis factor-a (TNF-a), interleukin (IL)-2, and IL-6. Hippocampi were weighted, homogenizied in phosphate buffer saline (PBS), centrifugated for 5 min at 5000xg and the supernatant was collected. Cytokine content, including IL-2, IL-6, and TNFα, and was determined using commercially available enzyme-linked immunosorbent assay (ELISA) kits (Interleukin 6 ELISA kits, Cat. No. WAR - 643 were purchased from Wkea (China), Interleukin 2 ELISA kits, Cat. No. MBS269718 were purchased from My Biosource (USA) and TNF α ELISA kits, Cat. No. WAR - 632 were purchased from Wkea (China)). Assays were performed according to the manufacturer's instructions. After the enzyme-substrate reaction was terminated, the optical density was measured at a wavelength of 450 nm. The concentration of IL-2, IL-6, and TNFα in the samples is then determined by comparing the O.D of the samples to the standard curve. The results were averaged and expressed as nanograms per liter ^4^.

**Evaluation of hippocampal oxidative stress biomarkers:**

1. **Measurement of Lipid peroxidase (Malondialdehyde):** Thiobarbituric acid (TBA) reacts with malondialdehyde (MDA) in acidic medium at temperature of 95°C for 30 min to form thiobarbituric acid reactive product the absorbance of the resultant pink product can be measured at 532 nm ^5^.( Lipid peroxide (Malondialdehyde) kit, Cat. NO. MD 25 29 (Biodiagnostics, Egypt)
2. **Measurement of Superoxide dismutase (SOD):** This assay relies on the ability of the enzyme to inhibit the phenazine methosulphate-mediated reduction of nitroblue tetrazolium dye ^6^. (Superoxide Dismutase (SOD) kit, Cat. No. SD 25 21 (Biodiagnostics, Egypt)
3. **Measurement of Total Antioxidant Capacity (TAC):** The determination of the antioxidative capacity is performed by the reaction of antioxidants in the sample with a defined amount of exogenously provide hydrogen peroxide (H202). The antioxidants in the sample eliminate a certain amount of the provided hydrogen peroxide. The residual H202 is determined colorimetrically by an enzymatic reaction which envolves the conversion of 3, 5, dichloro —2— hydroxyl benzensulphonate to a colored product ^7^. (Total antioxidant capacity kit, Cat. No. TA 25 13 (Biodiagnostics, Egypt)

**References**

1. Lin, H. B. *et al.* Memory deficits and neurochemical changes induced by C-reactive protein in rats: Implication in Alzheimer’s disease. *Psychopharmacology (Berl).* **204**, 705–714 (2009).

2. Suk-Chul, S. & Medicines, L. D.-U.-C. journal of natural. Ameliorating effect of new constituents from the hooks of Uncaria rhynchophylla on scopolamine-induced memory impairment‏. *Chin. J. Nat. Med.* **11**, 391-395. (2013).

3. Sun, M., Shen, X. & Ma, Y. Rehmannioside A attenuates cognitive deficits in rats with vascular dementia (VD) through suppressing oxidative stress, inflammation and apoptosis. *Biomed. Pharmacother.* **120**, 109492 (2019).

4. Afshari, J. T. *et al.* Determination of interleukin-6 and tumor necrosis factor-alpha concentrations in Iranian-Khorasanian patients with preeclampsia. *BMC Pregnancy Childbirth* **5**, 1–5 (2005).

5. Bakacak, M. *et al.* Changes in copper, Zinc, and malondialdehyde levels and superoxide dismutase activities in pre-eclamptic pregnancies. *Med. Sci. Monit.* **21**, 2414–2420 (2015).

6. Mandal, N., Mandal, S., Hazra, B., Sarkar, R. & Biswas, S. Assessment of the antioxidant and reactive oxygen species scavenging activity of methanolic extract of caesalpinia crista leaf. *Evidence-based Complement. Altern. Med.* **2011:17376**, (2011).

7. Samahy, M. H. El *et al.* Relation between carotid intima media thickness and oxidative stress markers in type 1 diabetic children and adolescents. *J. Diabetes Metab. Disord.* **12**, 1–7 (2013).
